# Effect of yoga or physical exercise on physical, cognitive and emotional measures in children: a randomized controlled trial

**DOI:** 10.1186/1753-2000-7-37

**Published:** 2013-11-07

**Authors:** Shirley Telles, Nilkamal Singh, Abhishek Kumar Bhardwaj, Ankur Kumar, Acharya Balkrishna

**Affiliations:** 1 Patanjali Research Foundation, Patanjali Yogpeeth, Haridwar, 249405 Uttarakhand, India

**Keywords:** Yoga, Physical exercise, Physical fitness, Cognitive performance, Self-esteem, School children

## Abstract

**Background:**

Previous studies have separately reported the effects of physical exercise and yoga in children, showing physical, cognitive and emotional benefits.

**Objectives:**

The present randomized controlled trial assessed the effects of yoga or physical exercise on physical fitness, cognitive performance, self-esteem, and teacher-rated behavior and performance, in school children.

**Methods:**

98 school children between 8 to 13 years were randomized as yoga and physical exercise groups {n = 49 each; (yoga: 15 girls, group mean age 10.4 ± 1.2 years), (physical exercise: 23 girls, group mean age 10.5 ± 1.3 years)}. Both groups were blind assessed after allocation, using: (i) the Eurofit physical fitness test battery, (ii) Stroop color-word task for children, (iii) Battle’s self-esteem inventory and (iv) the teachers’ rating of the children’s obedience, academic performance, attention, punctuality, and behavior with friends and teachers. After assessments the yoga group practiced yoga (breathing techniques, postures, guided relaxation and chanting), 45 minutes each day, 5 days a week. During this time the physical exercise group had jogging-in-place, rapid repetitive movements and relay races or games. Both groups were assessed at the end of 3 months. Data were analyzed with RM ANOVA and *post-hoc* tests were Bonferroni adjusted.

**Results:**

There was one significant difference between groups. This was in social self-esteem which was higher after physical exercise compared to yoga (p < 0.05). All the changes reported below are based on after-before comparisons, within each group. Both groups showed an increase in BMI, and number of sit-ups (p < 0.001). Balance worsened in the physical exercise group, while plate tapping improved in the yoga group (p < 0.001). In the Stroop task both groups showed improved color, word- and color-word naming (p < 0.01), while the physical exercise group showed higher interference scores. Total, general and parental self-esteem improved in the yoga group (p < 0.05).

**Conclusion:**

Yoga and physical exercise are useful additions to the school routine, with physical exercise improving social self-esteem.

**Trial registration:**

The study was registered in the Clinical Trials Registry of India (CTRI/2012/11/003112).

## Background

Schools have an important role in the development of children by identifying those with low physical fitness and by promoting health behaviors such as encouraging children to be active [1]. The most obvious benefits of physical exercise in children are improvements in physical fitness, which was shown in a study on 57 children [2]. Following seven weeks of exercise there were improvements in a fitness test, agility, counter movement jump test, sprint, systolic blood pressure, the fitness test, and fat percentage reduction.

A similar benefit has been demonstrated in other studies as well [3]. Apart from physical fitness there is evidence [4] that exercise influences cognitive function. A positive relationship between physical activity and cognitive and academic performance in school aged children was reported in a meta-analysis [5]. Also aerobic fitness in children is associated with higher measures of neuroelectric responsiveness (P3 in brain evoked potentials), faster cognitive processing speed [6] and better performance in a test of executive control [7].

In the preceding paragraphs, the benefits of physical exercise for physical fitness and cognition were described in pre-adolescents. Physical exercise is also associated with a positive effect on depression, anxiety, mood, self-esteem and higher academic performance [1]. These findings were supported by a study on 540 elementary school children [8], who were randomly assigned to a physical exercise program or a control condition during one academic year. Sub-population analysis showed that physical exercise had a positive effect on psycho-social Quality of Life (QoL) especially in urban and over-weight students. There was little effect of the physical exercise program on QoL overall.

These findings suggest that in addition to improving physical fitness and cognition, physical exercise appears to influence the psycho-social quality of life in children.

Another intervention which has positive effects on physical fitness, cognition and psycho-social wellbeing is yoga. Yoga is one of the components of ‘Be a Fit Kid’ which aims at improving physical exercise and nutrition in children [9]. Following the 12 week program, there was a significant improvement in body composition, fitness, nutrition knowledge, dietary habits and significant reductions in total cholesterol and triglyceride levels. This suggested that yoga based health promotion programs are well received by children and can favorably change being overweight and the development of adult life-style related diseases.

A study was conducted in 31 children between 7 and 12 years, who had bronchial asthma [10]. Sixteen children were assigned to a yoga program and 15 to a control group. Yoga was practiced three times per week for 7 weeks. Compared to the control group, the yoga group showed favorable outcomes in terms of muscular strength and endurance. After 2 weeks of home practice, yoga continued to improve BMI, flexibility, muscular strength and cardio-pulmonary fitness. Hence these two studies suggest the benefits of yoga in improving physical fitness in children.

Apart from the beneficial effects on physical fitness, yoga practice improves several aspects of cognition and executive functions. Executive functions are good predictors of math and reading competence throughout the school years [11,12]. It is possible that yoga might help improve executive functions [13], possibly related to the fact that yoga includes several mental techniques apart from the physical [14]. Also, school children practicing yoga for 10 days improved spatial memory scores [15], strategic planning [13] and the ability to concentrate [16]. Hence, yoga practice appears to influence physical fitness and cognitive functions. Apart from this yoga practice influences the emotional state [17]. School children were allocated to two after-school programs. One program offered yoga for 12 weeks while the other program did not. Self Worth and physical appearance were the primary outcome measures. Secondary outcomes included (i) perceptions of physical health and yoga teaching and (ii) focusing/relaxation. Controlling for pre-intervention well-being differences, children in the yoga group had better post-intervention negative behavior scores and balance than the non yoga group. The majority of children in the yoga group reported enhanced wellbeing. The results suggest a possible role of yoga as a preventive technique as well as a means of improving children’s perceived wellbeing. This was particularly important as the sample was drawn from inner-city children.

A separate report showed that mindfulness based approaches may improve adjustment among stressed and disadvantaged youth by improving self-regulatory capacities [18]. A pilot randomized controlled trial assessed the flexibility, acceptability and preliminary outcomes of a school based mindfulness and yoga intervention on 97 children who were randomized to the intervention condition (n = 51) and a control condition (n = 46). After 12 weeks the findings suggest that the intervention had a positive impact on problematic responses to stress, including rumination, intrusive thoughts and emotional arousal.

In the present study assessments were selected to simultaneously evaluate (i) physical fitness with the Eurofit fitness test battery [19], (ii) cognitive mechanisms related to attentional vitality and flexibility and volitional control over the neuropsychological functions which are involved in both word and color naming responses using the Stroop task and (iii) self-esteem, as a study conducted on children in India demonstrated that low self esteem is associated with several other mal-adaptations [20].

Previously both yoga and physical exercise have been separately found to influence the physical fitness, cognitive functioning and emotional wellbeing. Yoga and physical exercise differ in three main ways, since yoga practice places an emphasis on (i) breath awareness, (ii) regulated breathing, and (iii) conscious relaxation [14]. Hence the present randomized controlled trial aimed to compare the effects of yoga with those of physical exercise on physical fitness, cognitive functions and self-esteem.

Hence the hypothesis of the present study was that physical fitness, cognitive functions and self-esteem would change with yoga and with physical exercise, though the changes could be different based on the differences between the two, cited above when both interventions were separately included in the school day and children were followed up over a three month period.

## Methods

### Participants

In this study ninety-eight school children whose ages ranged between 8 and 13 years (group mean ± S.D., 10.5 ± 1.3 years) were selected as participants. Out of them 38 were females. Statistical calculation of the sample size was not done prior to the experiment. However *post-hoc* analyses showed that for the present study, with the sample size as 49 in each group, and with the Cohen’s d of 0.26 (small) the power calculation has been based on social self-esteem which was significantly different between groups in the RM ANOVA and *post-hoc* analysis comparing the after values of yoga and physical exercise groups [21]. The power (comparing the before-after yoga data) was 0.6969. All the participants were studying in a primary school which was randomly selected among schools in Haridwar, India. The inclusion criteria were: (i) participants of both sexes, studying in a school near the yoga center, (ii) those who were willing to follow the study conditions and (iii) those who were studying in grades 3 to 7 (age range 8 to 13 years for these grades). Exclusion criteria were (i) any physical or mental illness, or being on medication, based on a routine case history and medical examination, and (ii) color blindness based on the Ishihara test. None of the participants had to be excluded for these reasons. The participants were recruited with prior approval from the Principal of the school. The baseline characteristics of the two groups are given in Table 1.

**Table 1:** Baseline characteristics of yoga and physical exercise groups

**Groups**	**Yoga**	**Physical exercise**
**Age in years**	10.4 (1.2)	10.5 (1.3)
**Age range (years)**	8 -12	8 -13
**Gender ratio (B:G)**	34:15	26: 23
**Socio economic status**	Lower middle class	Lower middle class
**B.M.I. (kg/m**^**2**^**)**	15.10 (1.69)	15.36 (2.51)
**Right hand grip strength (kg)**	15.67 (5.30)	15.19 (6.00)
**Left hand grip strength (kg)**	15.06 (4.98)	14.31 (5.73)

B = Boys; G = Girls.

Values are Group Mean (S.D.).

Students of both groups (i) belonged to an urban location, (ii) their socio-economic status was categorized as lower middle class [22], with an average annual income of Indian Rupees 3,40,000 and (iii) the primary language spoken at their homes was not English.

The study was approved by the Institution’s Ethics Committee (Patanjali Research Foundation Ethics Committee). Signed informed consent was taken from the Principal of the school who informed the parents about the study. The parents gave their informed consent after receiving the information from the Principal of the school. The study was registered in the Clinical Trials Registry of India (CTRI/2012/11/003112).

### Design

**Figure 1:**
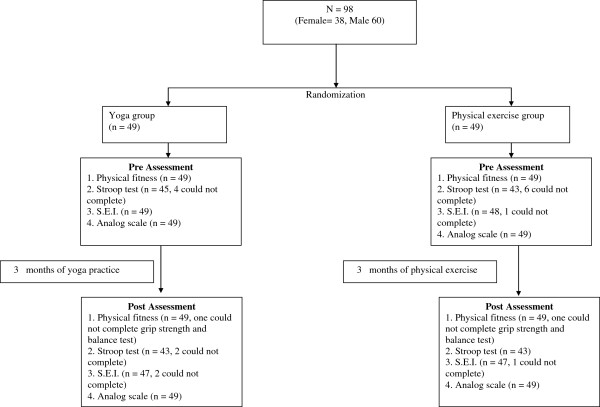
Trial profile for the RCT.

The 98 participants were randomized as two groups as follows: (i) Each participant was given a serial number from 1 to 98, which did not depend on their order of enrollment, their surname or any other factor. (ii) A specific computer program [23] was used to generate 98 random numbers. (iii) The 98 random numbers were written beside the serial numbers. Hence each participant was assigned a random number. (iv) The random numbers were written on identical slips of paper, folded identically. (v) A person who had no other part in the trial placed the slips of paper alternately in two boxes, one labeled ‘A’ and the other ‘B’. (vi) Persons in the ‘A’ group were allocated to Yoga and the persons in the B group were allocated to physical exercise. The final number in each group is mentioned in the Trial Profile (Figure 1). Through this method of randomization both groups were allocated 49 participants each. Hence the study is a parallel group design with allocation ratio of 1:1. The participants were recruited in August, 2010, and post data assessment was completed in December, 2010.

### Assessments

The participants were assessed for (i) physical fitness, (ii) performance in the Stroop task, (iii) self-esteem and (iv) analog scales, rated by the teachers. The primary outcome measures were (i) the Eurofit physical fitness test battery, (ii) the Stroop color-word naming task, and (iii) self-esteem. The secondary outcome measures were the teacher- reported (a) obedience, (b) academic performance, (c) attention, (d) punctuality, (e) behavior with friends, and (f) behavior with teachers.

#### Physical fitness based on the Eurofit battery [19]

##### Anthropometry

Height was measured to the nearest centimeter using a non-stretchable measuring tape (Gülick Anthropometric tape 60″ Model J00305, Lafayette Instrument, U.S.A.). The participants were instructed to remove their footwear and to stand in an upright position with their feet together. Weight was measured to the nearest 0.05 kg using an electronic balance. Participants were requested to remove heavy clothing and to stand straight. The body mass index (BMI) was calculated as the body weight (in kg) without shoes and light clothing, divided by the height (in m) squared.

##### Flamingo balance test

During the Flamingo Balance test the participants balanced on their preferred leg with the free leg flexed at the knee and their foot held close to the buttocks. The participants were supported by holding the hand of the instructor to reach the final position. The starting time was recorded with a stop watch as soon as the participant stopped taking support of the instructor. Number of falls in 60 seconds was recorded for every participant for the preferred (right) and non-preferred (left) leg separately.

##### Plate tapping test

In the plate tapping test two yellow discs 20 cm in diameter were placed on a flat brown surface with their centers 60 cm apart. In between the two discs a white rectangle (30 × 20 cm) was placed. The participants were asked to place the non-preferred hand on the rectangle and move the preferred hand back and forth between the two yellow discs over the rectangle. Two taps was counted as one cycle and the participants were asked to complete 25 cycles as fast as possible and the time taken to complete the 25 cycles was counted using a stopwatch. Each participant performed the test twice and the best performance was recorded.

##### Standing broad jump

For this test the initial line was marked with a tape on a non-skid soft mat. The participants were instructed to stand behind the initial line with their feet slightly apart and to jump forward at the word “Go” by slightly bending their knees and swinging their arms. They were also asked to land with both feet without falling backwards. The distance from the initial line to the back of their heels was measured. This process was repeated thrice for each participant and the longest distance out of the three attempts was recorded.

##### Handgrip test

Handgrip strength was measured using a hand grip dynamometer (Lafayette Instrument, Model 7498–05, U.S.A.). For this the participants held the dynamometer with their arm at right angles to the trunk of the body and their elbow touching the side of the body. The participants were instructed to squeeze the dynamometer with maximum isometric strength and maintain it for 5 seconds. Each hand was tested in three trials alternately, spaced 10 seconds apart. All participants were right hand dominant based on their response to a standard handedness inventory. For each hand the best value obtained of three trials was used for analysis.

##### Trunk strength

The participants were instructed to lie on their back with their knees bent at right angles and their feet flat on the floor which were held down by another participant. Their hands were kept crossed over their chest with their palms on opposite shoulders. At the word ‘Go’ the participants raised their upper body to a vertical position and then returned to the initial position. This was counted as one sit-up and the number of sit-ups in 30 seconds was noted using a stop-watch.

##### Bent arm hang

Bent arm hang was assessed using a horizontal bar suspended 192 cm above the ground. With the help of an instructor the participants grasped the horizontal bar with their palms facing away from the body and their chin was kept at the level of the bar. The participants were asked to hold this position as long as possible. The test started when the instructor released the participant and ended when the chin of the participant fell below the level of the horizontal bar or their head tilted backwards. The time from beginning to the end of the test was recorded with a stop watch.

##### 10 × 5 meter shuttle run

Two colored tapes were fixed 10 meters apart and the participants were requested to stand behind the initial line with one leg forward. At the word 'Go' the participants started running to the other tape, crossed it and then ran back to the initial line. This was continued for five times without stopping in between and the time taken to complete five rounds was recorded with a stopwatch.

#### Cognitive function

The children’s version of the Stroop color and word test [24] was used to assess cognitive function of the participants. The Stroop task measures volitional control over the neuropsychological functions which are involved in both word and color naming responses. We tested the inter-rater reliability with two separate individuals who were involved in the Stroop task assessment on the children. Inter-rater reliability assessment for 20 adult volunteers and correlation coefficients of 0.84 (word score), 0.80 (color score) and 0.60 (color-word score) were obtained, suggesting an adequate inter-rater reliability. The test was in the form of a booklet which contains 3 pages. The first page tests how fast the participant can read words, the second page tests how fast the participants can name the colors on the page, and in the third page the participants were asked to name the color of the ink the words were printed in, ignoring the word that was printed for each item. The task was administered individually. For any mistake the participants were asked to stop and proceed after correcting the mistake. The participants were given 45 seconds for each page. Detailed instructions were given to the participants before starting the test. A stop-watch was used to record the time taken to complete the task.

#### Self-esteem

Self-esteem of the participants was measured using the Indian adaptation of Battle’s self-esteem questionnaire. The reliability of the questionnaire has been established for use with Indian children [25]. The questionnaire has 50 close-ended questions with 4 subscales. The subscales were (i) general self-esteem, (ii) social self-esteem, (iii) academic self-esteem, and (iv) parental self-esteem. There are 20 items on general self-esteem, 10 items on social self-esteem, 10 items on academic self-esteem, and 10 items on parental self-esteem. The test was administered in a group. The participants were given instructions to attempt all the questions, and to complete filling in the questionnaire in the allotted time i.e., 15 minutes, and to ask the instructor if they were not able to understand any question.

#### Analog scales

The teachers’ ratings of the (i) obedience, (ii) academic performance, (iii) attention, (iv) punctuality, (v) behavior with friends, and (vi) behavior with teachers were assessed for each participant using six separate visual analog scales. Each analog scale was a 10 centimeter long doubly anchored scale, with one end (score = 10) of the scale indicating the highest score while the other end (score = 0) indicated the lowest score. There was a separate scale for each of the six variables. Teachers were requested to place a vertical mark on the horizontal line to indicate the level of their rating. For each individual the score for a particular quality assessed was obtained by measuring the distance in millimeters from the end of the line where the score was ‘0’ upto the mark made by the teachers. All the analog scales were scored in one direction (i.e., with ‘0’ on the left). Separate analog scales were provided for each of the six variables. The teachers were requested to place a vertical mark on the horizontal line wherever they felt appropriate for each student.

### Interventions

Interventions were given for three months for five days in a week and on each day the participants practiced either yoga or physical exercise for 45 minutes. Yoga practice involved *pranayamas* (yoga breathing techniques), *sithilikarna vyayama* (loosening exercises), *asanas* (physical postures), chanting and yoga relaxation techniques. Details of the yoga practice are given in Table 2.

**Table 2:** Details about the yoga program mentioning a three day sequence which was repeated throughout the three months

**Day 1**			**Day 2**		**Day 3**	
**Sl. no.**	**Yoga practice**	**Duration**	**Yoga practice**	**Duration**	**Yoga practice**	**Duration**
**1.**	**Start:**	2 min.	**Start:**	2 min.	**Start:**	2 min.
	*Sukhasana* (Easy posture) *+ Gayatri Mantra*		*Padmasana* (Lotus posture) *+ Mahamrityunjaya Mantra*		*Siddhasana* (Perfect posture) *+ Prarthana Mantra*	
**2. (i)**	***Pranayamas:***	180 strokes/ 3 min.	***Pranayamas:***	180 strokes/ 3 min.	***Pranayamas:***	180 strokes/ 3 min.
	*Kapalabhati* (High frequency yoga breathing)		*Kapalabhati* (High frequency yoga breathing)		*Kapalabhati* (High frequency yoga breathing)	
**(ii)**	*Bhastrika* (Bellows breathing)	36-50 strokes/ 3 min.	*Bhastrika* (Bellows breathing)	36-50 strokes/ 3 min.	*Bhastrika* (Bellows breathing)	36-50 strokes/ 3 min.
**(iii)**	*Ujjayi* (Victorious breathing)	5-10 rounds/ 3 min.	*Ujjayi* (Victorious breathing)	5-10 rounds/ 3 min.	*Ujjayi* (Victorious breathing)	5-10 rounds/ 3 min.
**3.**	***Surya Namaskara *** **(Sun salutation)**	4 rounds/ 4 min.	***Surya Namaskara *** **(Sun salutation)**	6 rounds/ 4 min.	***Surya Namaskara *** **(Sun salutation)**	8 rounds/ 4 min.
**4.(i)**	***Pranayamas:***	12-15 rounds/ 3 min.	***Pranayamas:***	12-15 rounds/ 3 min.	***Pranayamas:***	12-15 rounds/ 3 min.
	*Anulom-vilom* (Alternate nostril breathing)		*Anulom-vilom* (Alternate nostril Breathing)		*Anulom-vilom* (Alternate nostril Breathing)	
**(ii)**	*Bhramari* (Bumble bee breathing)	10-15 rounds/ 3 min.	*Bhramari* (Bumble bee breathing)	10-15 rounds/ 3 min.	*Bhramari* (Bumble bee breathing)	10-15 rounds/ 3 min.
**(iii)**	*Udgeeth* (OM chanting)	8-10 rounds/ 3 min.	*Udgeeth* (OM chanting)	8-10 rounds/ 3 min.	*Udgeeth* (OM chanting)	8-10 rounds/ 3 min.
**5.**	***Asanas:***		***Asanas:***		***Asanas:***	
**(i)**	**Sitting postures:**	5 min.	**Sitting postures:**	5 min.	**Sitting postures:**	5 min.
	Butterfly Pose (for warm up), *Padmasana* (Lotus posture) *, Vajrasana* (Diamond posture)*,Sasankasana* (Rabbit posture) *, Singhasana* (Lion posture)		Butterfly Pose (for warm up), *Ardha Ustrasana* (Half camel posture), *Gomukhasana* (Cow’s face posture), *Paschimottanasana* (Back stretching posture)*, Singhasana* (Lion posture)		Butterfly Pose (for warm up), *Ardha Matsyendrasana* (Half spinal twist) (both sides) *, Gomukhasana* (Cow’s face posture)*, Singhasana* (Lion posture)	
**(ii)**	**Prone postures:**	4 min.	**Prone postures:**	4 min.	**Prone postures:**	4 min.
	*Makarasana* (Crocodile posture)*, Balasana* (Child posture)*, Bhujaangasana* (Cobra posture)*, Dhanurasana* (Bow posture)		*Balasana* (Child posture)*, Bhujaangasana* (Cobra posture)*, Dhanurasana (Bow posture) Salabhasana* (Locust posture)		*Makarasana* (Crocodile posture)*, Bhujaangasana* (Cobra posture)*, Dhanurasana* (Bow posture)*, Mayurasana* (Peacock posture)	
**(iii)**	**Supine postures:**	4 min.	**Supine postures:**	4 min.	**Supine postures:**	4 min.
	*Uttana padasana* (Raised legs posture)*, Pavanamuktasana, Naukasana* (Boat posture)		*Uttana padasana* (Raised legs posture)*, Pavanamuktasana Sarvangasana* (Shoulder stand posture)*, Halasana* (Plough posture)		*Uttana padasana* (Raised legs posture)*, Naukasana* (Boat posture)*, Halasana* (Plough posture)*, Chakrasana* (Wheel posture)	
**(iv)**	**Standing postures:**	5 min.	**Standing postures:**	5 min.	**Standing postures:**	5 min.
	(side bending and twisting as warm up) *Tadasana* (Palm tree posture)*, Tiryak Tadasana* (Swaying palm tree posture)*, Vrikshasana* (Tree posture)*, Garudasana* (Eagle posture)*, Konasana* (Angle posture)		(side bending and twisting as warm up) *Tadasana* (Palm tree posture)*, Tiryak Tadasana* (Swaying palm tree posture), *Ardha chakrasana* (Half wheel posture)*, Padhastasana* (Forward bending posture)*, Konasana* (Angle posture)		(side bending and twisting as warm up) *Tadasana* (Palm tree posture)*, Tiryak Tadasana* (Swaying palm tree posture) *Veerbhadrasana* (Warrior posture)*, Ardha chakrasana* (Half wheel posture)*, Padhastasana* (Forward bending posture	
**6.**	**For relaxation:**	3 min.	**For relaxation:**	3 min.	**For relaxation:**	3 min.
	*Savasana* (Corpse posture)*, Hasyasana* (Laughter yoga)		*Yoganidra* (Yogic sleep)*, Hasyasana* (Laughter yoga)		*Savasana* (Corpse posture)*, Hasyasana* (Laughter yoga)	
**Total timings**		45 minutes	**Total timings**	45 minutes	**Total timings**	45 minutes

Intervention of the physical exercise group involved jogging in place, rapid bending forward and backward, bending sideways, spinal twisting and relay races or games.

The three differences between yoga and physical exercise are: (i) yoga places an importance on awareness, (ii) on relaxation and (iii) on breath regulation. Details of the physical exercise program are given in Table 3. Both yoga and physical exercise were conducted during school hours. All participants were in one yoga class and similarly all participants were in one physical exercise class. The classes were taught by trained instructors who had completed 17 years of education, i.e., they were post graduates and had a master’s degree. The instructors who took yoga and physical exercise were not school teachers. They were part of the yoga institution which conducted the trial. Both of them had approximately two years training in yoga and six months in teaching yoga. One of them, chosen randomly was asked to teach the physical exercise class along with a school teacher who had training in physical exercise. Class attendance was monitored by one of the class teachers.

**Table 3:** Physical exercise group

**Sl. no.**	**Name of exercise**	**Timing for each exercise (in minutes)**	**Recovery time (in minutes) (without instruction)**
**1**	**Jogging in place**	**8**	**1**
	(i) Slow jogging		
	(ii) Thighs perpendicular to the trunk		
	(iii) Knees flexed, feet directed sideways		
	(iv) Knees flexed, heels touching the buttocks		
**2**	**Rapid bending forwards & backwards**	**5**	**1**
	(i) With legs together		
	(ii) With legs apart		
**3**	**Bending sideways**	**5**	**1**
	(i) With legs together		
	(ii) With legs apart		
**4**	**Spinal twisting**	**3**	**1**
**5**	**Relay races/games**	**18**	**2**
	**Total time of intervention**	45 minutes	

### Data extraction

Scoring of all assessments was carried out by an individual who was blinded to which group the participants belonged.

#### Physical fitness based on the Eurofit battery

For this category the scores were directly used for analysis.

#### Cognitive function

Scoring the Stroop test gives three types of raw scores (i) raw word scores, (ii) raw color scores, and (iii) raw color-word scores. To get the pure interference score of the color-word page independent of the participants’ reading or color naming ability, interference raw scores (I) were derived by subtracting color raw scores from color-word raw scores. T-scores (according to age) were calculated for all the raw scores based on the normative data given in the manual.

#### Self-esteem

The self-esteem scale was binomial. In the manual it is mentioned which responses should be scored as ‘1’ depending on whether the response was “No” or “Yes”. If the responses did not follow this pattern items were scored as ′0‵. Total self-esteem was calculated by adding the scores of all the subscales.

#### Analog scales

The score of each participant was obtained by measuring the distance from the left of the line (score = 0) up to the mark made by the teacher. The precision of measurement was 0.1 mm.

### Data analysis

Repeated measures analyses of variance (ANOVA) followed by *post-hoc* analyses with Bonferroni adjustment (corrected Bonferroni value of 0.025) were done to compare data after the two interventions with data recorded before the two interventions, using PASW Version 18.0. There was one Within subjects factor i.e., States (pre and post) and one Between subjects factor i.e., Groups (yoga and physical exercise).

## Results

The group mean values ± SD for the different variables are given in Tables 4, 5, 6 and 7.

**Table 4:** Variables of the EUROFIT physical fitness battery

**Variables**	**n**	**Yoga (n = 49)**			**Physical exercise (n = 49)**		
		**Pre**	**Post**	**Cohen’s d**	**Pre**	**Post**	**Cohen’s d**
**B.M.I. (kg/m**^**2**^**)**	49	15.10 (1.69)	16.34 (1.88)***	0.6936	15.36 (2.51)	16.60 (2.78)***	0.4681
**Bent arm (seconds)**	49	3.64 (4.2)	2.60 (4.32)	0.2441	3.99 (8.84)	3.29 (6.63)	0.0895
**Plate tapping (seconds)**	49	17.98 (3.56)	16.37 (2.96)***	0.4917	17.46 (3.41)	16.85 (3.05)	0.1885
**Shuttle run (seconds)**	49	25.14 (2.47)	25.15 (2.20)	0.0042	26.09 (2.55)	25.44 (2.28)	0.2687
**Broad jump (cm)**	49	120.44 (28.18)	116.74 (20.95)	0.1490	111.41 (27.50)	112.58 (21.80)	0.0471
**No. of sit-ups in 30 sec**	49	9.61 (5.60)	12.18 (5.90)***	0.4468	8.63 (6.28)	11.37 (5.51)***	0.4638
**Right hand grip strength (kg)**	48	15.67 (5.30)	16.65 (5.54)	0.1807	15.19 (6.00)	15.67 (5.36)	0.0843
**Left hand grip strength (kg)**	48	15.06 (4.98)	15.88 (5.30)	0.1594	14.31 (5.73)	14.98 (5.73)	0.1169
**Flamingo balance right (no. of falls)**	48	1.63 (2.06)	2.50 (4.27)	0.2595	1.29 (1.81)	2.83 (4.26)**	0.4705
**Flamingo balance left (no. of falls)**	48	0.98 (1.50)	1.65 (3.42)	0.2537	1.56 (2.68)	2.19 (3.46)	0.2035

**p < .01, *post-hoc* analyses with Bonferroni adjustment; ***p < .001, *post-hoc* analyses with Bonferroni adjustment compared with pre. Values are group mean (S.D.).

**Table 5:** Values for the Stroop color-word task

**Variables**	**Yoga (n = 43)**			**Physical exercise (n = 43)**		
	**Pre**	**Post**	**Cohen’s d**	**Pre**	**Post**	**Cohen’s d**
**Word raw score**	63.63 (18.77)	68.70 (21.26)**	0.2528	61.60 (18.94)	68.65 (18.14)***	0.3801
**Word T score**	48.70 (9.97)	51.33 (11.47)**	0.2447	47.30 (10.25)	51.09 (9.75)***	0.3788
**Color raw score**	43.86 (9.41)	48.63 (9.21)***	0.5123	43.09 (9.58)	50.02 (9.83)***	0.7140
**Colore T score**	42.12 (6.00)	44.47 (5.85)**	0.3965	41.21 (6.21)	45.88 (6.18)***	0.7538
**Color-word raw score**	25.77 (6.98)	29.30 (8.43)**	0.4561	27.88 (7.49)	30.86 (6.97)**	0.4119
**Color-word T score**	38.35 (8.28)	42.60 (9.48)**	0.4775	40.88 (8.34)	44.93 (7.66)**	0.5057
**Interference raw score**	−18.09 (7.60)	−19.56 (8.43)	0.1831	−15.21 (8.17)	−19.35 (7.34)**	0.5330
**Interference T-score**	46.09 (10.26)	48.23 (8.09)	0.2344	43.28 (10.30)	47.88 (8.11)*	0.4962

*p < .05, *post-hoc* analyses with Bonferroni adjustment; **p < .01, *post-hoc* analyses with Bonferroni adjustment; ***p < .001, *post-hoc* analyses with Bonferroni adjustment compared with pre. Values are group mean (S.D.).

**Table 6:** Values for the Indian adaptation of Battle’s self esteem inventory

**Variables**	**Yoga (n = 47)**			**Physical exercise (n = 47)**		
	**Pre**	**Post**	**Cohen’s d**	**Pre**	**Post**	**Cohen’s d**
**Total Score of S.E.**	33.51 (6.46)	36.98 (4.82)***	0.6088	35.13 (5.62)	36.74 (5.40)	0.2921
**General S.E**	11.68 (2.83)	13.68 (2.24)***	0.7836	12.36 (2.79)	13.28 (2.71)	0.3345
**Social S.E**	5.62 (1.49)	6.06 (1.76)	0.2698	6.43 (1.51)	6.77 (1.56)	0.2214
**Academic S.E**	7.89 (1.82)	8.28 (1.21)	0.2523	7.91 (1.77)	8.02 (1.64)	0.0644
**Parental S.E.**	8.32 (2.05)	8.96 (1.38)*	0.3662	8.43 (1.33)	8.68 (1.35)	0.1865

Values are group mean (S.D.).

*p < .05, *post-hoc* analyses with Bonferroni adjustment; ***p < .001, *post-hoc* analyses with Bonferroni adjustment compared with pre.

**Table 7:** Values for the analog scales rated by the teachers

**Variables**	**Yoga (n = 49)**			**Physical exercise (n = 49)**		
	**Pre**	**Post**	**Cohen’s d**	**Pre**	**Post**	**Cohen’s d**
**Obedience**	6.05 (2.36)	7.59 (1.46)***	0.7847	5.98 (2.12)	7.68 (1.23)***	0.9808
**Academic performance**	5.25 (2.32)	7.48 (1.85)***	1.0628	5.13 (2.72)	7.733 (1.33)***	0.2158
**Attention**	5.48 (2.27)	7.42 (1.19)***	1.0704	5.32 (2.50)	7.71 (1.41)***	1.1776
**Punctuality**	6.28 (2.23)	7.87 (1.50)***	0.8366	6.23 (2.72)	7.94 (1.36)***	0.7952
**Behavior with friends**	6.61 (1.89)	7.78 (1.04)***	0.7670	6.53 (1.84)	7.86 (1.09)***	0.8794
**Behavior with teachers**	7.16 (1.67)	8.35 (1.19)***	0.8206	7.01 (1.68)	8.39 (0.98)***	1.0034

Values are group mean (S.D.).

***p < .001, *post-hoc* analyses with Bonferroni adjustment compared with pre.

### Repeated measures analyses of variance (RMANOVA)

**Figure 2:**
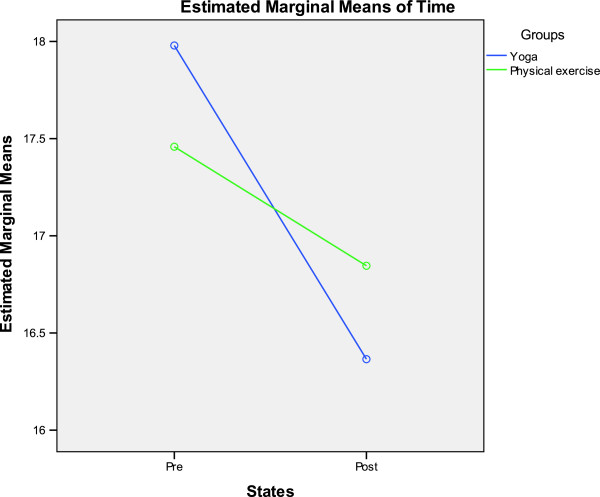
Graphical representation of the Groups*States interaction for the plate tapping task. Dependent variable (time taken for plate tapping in seconds) on the Y axis, one of the independent variables (States) on the X axis, and the other independent variable (Groups) as separate lines on the graph.

**Figure 3:**
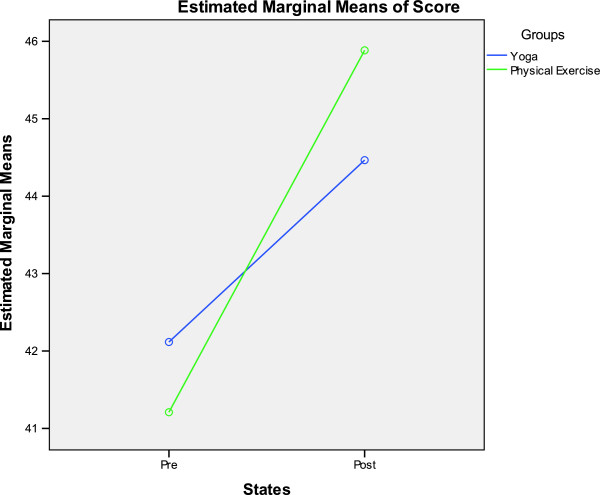
Graphical representation of the Groups*States interaction for the color T scores in the Stroop task. Dependent variable (scores) on the Y axis, one of the independent variables (States) on the X axis, and the other independent variable (Groups) as separate lines on the graph.

The ANOVA values for the Within-Subjects factor (States), Between-Subjects factor (Groups) and interaction between the two for the different variables for physical fitness, cognitive function, self-esteem and analog scales are provided in Tables 8, 9, 10 and 11 respectively. A significant interaction between Groups and States for any variable suggests that the two are interdependent. Groups * States interaction was significant for plate tapping and color T scores. This significant interaction has been graphically presented in Figures 2 and 3.

**Table 8:** ANOVA table for variables of the EUROFIT physical fitness battery

**Sl. no.**	**Factors**	**Variable**	**F**	**df**	**Huynh-Feldt ϵ**	**p**
**I**	**Within subjects (states)**	**B.M.I**	249.242	1,94	1	0.000
		**Bent arm hang**	1.605	1,94	1	0.208
		**Shuttle run**	1.204	1,96	1	0.275
		**Sit-ups**	29.569	1,96	1	0.000
		**Plate tapping**	20.870	1,94	1	0.000
		**Broad jump**	0.327	1,96	1	0.569
		**Flamingo balance Rt leg**	13.600	1,94	1	0.000
		**Flamingo balance Lt leg**	4.048	1,94	1	0.047
		**Grip strength Rt hand**	4.447	1,94	1	0.038
		**Grip strength Lt hand**	6.361	1,94	1	0.013
**II**	**Between subjects (groups)**	**B.M.I**	0.330	1,94	-	0.567
		**Bent arm hang**	0.201	1,94	-	0.655
		**Shuttle run**	2.598	1,94	-	0.251
		**Sit-ups**	0.701	1,94	-	0.867
		**Plate tapping**	0.001	1,94	-	0.974
		**Broad jump**	2.149	1,94	-	0.146
		**Flamingo balance Rt leg**	0.087	1,94	-	0.769
		**Flamingo balance Lt leg**	1.300	1,94	-	0.257
		**Grip strength Rt hand**	0.455	1,94	-	0.502
		**Grip strength Lt hand**	0.589	1,94	-	0.804
**III**	**Group × states**	**B.M.I**	0.011	1, 94 (Groups) × 94 (States)	-	0.917
		**Bent arm Hang**	0.28	1, 94 (Groups) × 94 (States)	-	0.868
		**Shuttle run**	1.335	1, 94 (Groups) × 94 (States)	-	0.251
		**Sit-ups**	0.028	1, 94 (Groups) × 94 (States)	-	0.867
		**Plate tapping**	4.222	1, 94 (Groups) × 94 (States)	-	0.043
		**Broad jump**	1.211	1, 94 (Groups) × 94 (States)	-	0.274
		**Flamingo balance Rt leg**	1.798	1, 94 (Groups) × 94 (States)	-	0.183
		**Flamingo balance Lt leg**	0.004	1, 94 (Groups) × 94 (States)	-	0.948
		**Grip strength Rt hand**	0.523	1, 94 (Groups) × 94 (States)	-	0.471
		**Grip strength Lt hand**	0.062	1, 94 (Groups) × 94(States)	-	0.804

**Table 9:** ANOVA table for the Stroop task

**Sl. no.**	**Factors**	**Variable**	**F**	**df**	**Huynh-Feldt ϵ**	**p**
**I**	**Within subjects**	**Word raw score**	25.025	1,84	1	0.000
		**Word T score**	22.336	1,84	1	0.000
		**Color raw score**	62.842	1,84	1	0.000
		**Color T score**	37.286	1,84	1	0.000
		**Color-word raw score**	19.517	1,84	1	0.000
		**Color-word T score**	23.838	1,84	1	0.000
		**Interference raw score**	7.923	1,84	1	0.006
		**Interference T score**	7.869	1,84	1	0.006
**II**	**Between subjects**	**Word raw score**	0.067	1,84	-	0.796
		**Word T score**	0.146	1,84	-	0.704
		**Color raw score**	0.027	1,84	-	0.870
		**Color T score**	0.047	1,84	-	0.828
		**Color-word raw score**	1.634	1,84	-	0.205
		**Color-word T score**	2.263	1,84	-	0.136
		**Interference raw score**	1.253	1,84	-	0.266
		**Interference T score**	0.985	1,84	-	0.324
**III**	**Group × states**	**Word raw score**	0.666	1, 84 (Groups) × 84 (States)	-	0.417
		**Word T score**	0.733	1, 84 (Groups) × 84 (States)	-	0.394
		**Color raw score**	2.141	1, 84 (Groups) × 84 (States)	-	0.146
		**Color T score**	4.088	1, 84 (Groups) × 84 (States)	-	0.046
		**Color-word raw score**	0.143	1, 84 (Groups) × 84 (States)	-	0.706
		**Color-word T score**	0.015	1, 84 (Groups) × 84 (States)	-	0.902
		**Interference raw score**	1.804	1, 84 (Groups) × 84 (States)	-	0.183
		**Interference T score**	1.051	1, 84 (Groups) × 84 (States)	-	0.308

**Table 10:** ANOVA for the Indian adaptation of Battle’s self esteem inventory

**Sl. no.**	**Factors**	**Variable**	**F**	**df**	**Huynh-Feldt ϵ**	**p**
**I**	**Within subjects**	**Academic self esteem**	1.950	1,92	1	0.166
		**General S.E.**	18.637	1,92	1	0.168
		**Parental S.E.**	6.857	1,92	1	0.069
		**Social S.E.**	4.292	1,92	1	0.041
		**Total S.E.**	19.583	1,92	1	0.000
**II**	**Between subjects**	**Academic self esteem**	0.167	1,92		0.683
		**General S.E.**	0.103	1,92	-	0.749
		**Parental S.E.**	0.097	1,92	-	0.756
		**Social S.E.**	8.016	1,92	-	0.006
		**Total S.E.**	0.475	1,92	-	0.493
**III**	**Group × states**	**Academic self esteem**	0.623	1, 92(states) × 92 (Groups)	-	0.432
		**General S.E.**	2.583	1, 92(states) × 92 (Groups)	-	0.111
		**Parental S.E.**	1.259	1, 92 (states) × 92 (Groups)	-	0.265
		**Social S.E.**	0.078	1, 92(states) × 92 (Groups)	-	0.780
		**Total S.E.**	2.595	1, 92(states) × 92 (Groups)	-	0.111

**Table 11:** ANOVA for analog scales rated by the teachers

**Sl. no.**	**Factors**	**Variable**	**F**	**df**	**Huynh-Feldt ϵ**	**p**
**I**	**Within subjects**	**Obedience**	76.418	1,96	1	0.000
		**Academic performance**	141.506	1,96	1	0.000
		**Attention**	132.197	1,96	1	0.000
		**Punctuality**	51.118	1,96	1	0.000
**II**	**Between subjects**	**Obedience**	0.001	1,96	-	0.975
		**Academic performance**	0.034	1,96	-	0.854
		**Attention**	0.027	1,96	-	0.869
		**Punctuality**	0.002	1,96	-	0.969
**III**	**Group × states**	**Obedience**	0.214	1, 96 (Groups) × 96 (States)	-	0.645
		**Academic performance**	0.856	1, 96 (Groups) × 96 (States)	-	0.357
		**Attention**	1.375	1, 96 (Groups) × 96 (States)	-	0.244
		**Punctuality**	0.069	1, 96 (Groups) × 96 (States)	-	0.794

### Post - hoc analyses

There was only one significant difference between the groups in social self-esteem which was higher in the physical exercise group and all other differences were not significant. Other differences mentioned were post-pre comparisons within a group.

#### Physical fitness based on the Eurofit battery

The results given below are post-pre comparisons within each group.

There was a significant increase in B.M.I. (p < .0001) with 95% Confidence Interval (CI) of [−1.012, -1.453] for yoga group; (p < .0001) with 95% CI of [−1.028, -1.469] for physical exercise group and in the number of sit-ups in 30 seconds (p = .0003) with 95% CI of [−1.202, -3.941] for yoga group; (p = .0001) with 95% Confidence CI of [−1.365, -4.104] for physical exercise group. Time taken to complete the plate tapping task reduced significantly in the yoga group alone (p < .0001) with 95% CI of [2.298, 0.930] while the number of falls during the flamingo balance test (preferred leg) increased significantly only in the physical exercise group (p = .001) with 95% CI of [−0.828, -2.922].

#### Cognitive function

The results given below are post-pre comparisons within each group.

The yoga and physical exercise groups showed a significant increase in word raw scores (p = .003) with 95% CI of [−1.664,-8.476]; (p < .0001) with 95% CI of [−3.641, -10.452], word T scores (p = .007) with 95% CI of [−0.718, -4.538]; (p = .0001) with 95% CI of [−1.881, -5.7], color raw scores (p < .0001) with 95% CI of [−2.692, -6.842]; (p < .0001) with 95% CI of [−4.855, -9.005], color T scores (p = .005) with 95% CI of [−0.731, -3.966]; (p < .0001) with 95% CI of [−3.057, -6.292], color-word raw scores (p = .001) with 95% CI of [−1.462, -5.608]; (p = .005) with 95% CI of [−0.904, -5.049] and color-word T scores (p = .001) with 95% CI of [−1.865, -6.647]; (p = .001) with 95% CI of [−1.655, -6.438]. The physical exercise group showed a significant increase in interference T scores (p = .008) with 95% CI of [−1.224, -7.985] and a significant reduction in interference raw scores (p = .004) with 95% CI of [6.939, 1.340].

#### Self-esteem

There was a significant difference in the after values between groups for social self-esteem which was higher in the physical exercise group (p < .05) with 95% CI of [−0.020, -1.385].

The remaining results given below are post-pre comparisons within the yoga group. There was a significant increase in total self-esteem (p < .0001) with 95% CI of [−1.854, -5.082], general self-esteem (p < .0001) with 95% CI of [−1.052, -2.948] and parental self-esteem (p = .01) with 95% CI of [−0.159, -1.118].

#### Analog scales

The results given below are after-before comparisons within each group.

In the yoga and physical exercise groups there was a significant improvement in obedience (p < .0001 for both groups) with 95% CIs of [−1.014, -2.055] for yoga group and [−1.186, -2.226] for the physical exercise group, academic performance (p < .0001 for both groups) with 95% CIs of [−1.657, -2.769] for yoga group and [−2.032, -3.172] for the physical exercise group, attention (p < .0001 for both groups) with 95% CIs of [−1.413, -2.468] for yoga group and [−1.854, -2.909] for the physical exercise group, punctuality (p < .0001 for both groups) with 95% CIs of [−0.938, -2.229] for yoga group and [−1.059, -2.350] for the physical exercise group, behavior with friends (p < .0001 for both groups) with 95% CIs of [−0.755, -1.584] for yoga group and [−0.916, -1.745] for the physical exercise group and behavior with teachers (p < .0001 for both groups) with 95% CIs of [−0.818, -1.561] for yoga group and [−1.002, -1.745] for the physical exercise group.

### Summary of the results

(i) The social self-esteem was the only variable which significantly differed between groups at post testing. (ii) The other changes were post-pre comparisons within each group. (iii) There were significant interactions between Groups and States (Groups * States) for (a) plate tapping time and (b) the Stroop task color T scores. (iv) Physical fitness: both groups increased BMI and number of sit-ups. The yoga group reduced time in the repetitive plate tapping task. The control group increased falls for the preferred leg in the balance test. (v) Stroop task: both groups showed an increase in word scores, color scores and color-word scores. The physical exercise group showed reduced interference raw scores and an increase in interference T scores. (vi) Self-esteem: See point (i) for between-group differences. The yoga group showed an increase in total, general and parental self-esteem. (vii) Analog scales: Both groups showed an improvement in obedience, academic performance, attention, punctuality, behavior with friends and behavior with teachers.

None of the children reported any adverse event or adverse reaction to the two interventions and the assessments, though this was not specifically asked to them. Here, the words adverse event and adverse reaction are based on descriptions provided by collaborating centers of the WHO International Drug Monitoring Center.

## Discussion

The present randomized controlled trial conducted on 98 school children showed a single between groups difference in social self-esteem after 3 months. All other differences were within group comparisons compared to the baseline.

The first set of variables assessed physical fitness using the Eurofit fitness battery. The factors influencing the results of the individual tests in the Eurofit fitness battery are described individually below.

The number of sit-ups in a fixed time (which increased in both groups) tests abdominal strength and muscular endurance [26]. The comparable improvement following yoga and physical exercise shows that both practices improve trunk strength and endurance. The plate tapping test (Reaction Tap Test) is a reaction test using an alternating tapping action which measures upper body reaction time, hand-eye quickness and coordination. The yoga group showed a decrease in the time needed to perform the task compared to the baseline, but not compared to the physical exercise group. The interaction between Groups and States was significant for this variable (Figure 2), hence the interpretation should be viewed with the knowledge that it may not be accurate and may even be misleading [27]. Previously yoga practice improved repetitive tapping performance in healthy volunteers [28] and in volunteers who used a computer keyboard for more than 5 hours a day [29]. Motor speed is determined by muscle strength, endurance and co-ordination [30]. Taking into account the limitations in interpreting the findings the decreased time taken in the yoga group suggests that yoga practice could increase these muscle functions.

The BMI significantly increased to a comparable extent in both yoga and physical exercise groups. There could be different reasons for this. Baseline assessments were carried out in August which coincides with summer while the final assessments were carried out in December, at the onset of winter. Extreme low temperatures and associated environmental conditions are associated with decreased plasma leptin levels and increased neuropeptide Y levels, which increase the appetite and result in a gain in body weight [31,32]. Apart from seasonal effects the increase in BMI could also be related to level of activity [33]. Since the change in BMI was observed in both groups and there was no control group, this could also be a time effect related to normal changes in growth and development, occurring naturally over three months.

The maintenance of body weight depends on the energy balance between weight gained and weight lost [34]. In the present study participants did increase their level of physical activity. However this could have been less than their appetite and energy intake, which could explain the net gain in BMI. Finally, among the assessments of the Eurofit Physical Fitness Test Battery, there was a significant decrease in balance in the physical exercise group. This single leg balance test assesses leg, pelvic and trunk strength. Previously yoga practice improved balance in normal volunteers [35] and in older adults [36]. The absence of change in the yoga group in the present study could be related to the fact that the yoga practice consisted of yoga postures and yoga breathing techniques practiced for the same duration of time, whereas earlier studies [35,36] emphasized yoga postures.

The Stroop task assesses attentional vitality and flexibility and makes use of the fact that it is possible to read words more quickly and automatically than naming colors [24]. The cognitive mechanisms involved in this task are called directed attention which means the attention should be inhibited or one response should be stopped in order to say or do something else.

A previous study on 74 children between 7 and 10 years of age showed that greater aerobic fitness was associated with better performance on each of the 3 Stroop conditions (word, color, color-word) independently of the other variables [7]. The improvement in word and color scores in the yoga and physical exercise groups suggests that like the study cited here [7], the improved scores may be due to better aerobic fitness. This could also apply to the color-word score which reflects directed attention. The increase in interference T scores in the physical exercise group suggests reduced flexibility and ability to respond to the task demands after three months. Color T scores also showed significant interaction between Groups and States suggesting that attempting to interpret the post-pre changes could be inaccurate and even be misleading [27].

Self-esteem is related to the evaluative judgments children make about their characteristics and qualities, including their attitude about themselves and their sense of worthiness [37]. The social self-esteem increased after physical exercise compared to yoga. Social self-esteem is the aspect of self-esteem that refers to an individual’s perception of and feelings about the quality of their relationships with peers [38]. Positive self-esteem is indicative of a positive and integral personal and social identity [38]. The yoga group showed an improvement in general, parental and total self-esteem in a post-pre comparison. This could be related to the fact that yoga practice increases emotional resilience [39].

The teachers’ reports showed improvements in both groups after 3 months compared to the respective baselines. Teachers’ ratings of childrens’ behavior are an essential tool of psychological research and practice [40]. Also, a teacher’s perceptions of a child’s behavior predicted aspects of the teacher’s behavior towards the child, even after accounting for the child’s behavior. Hence these reports were also considered when evaluating the effects of the two interventions.

In summary, both yoga and physical exercise practiced over a three month period showed significant improvements in tests for physical fitness, the Stroop task and assessments made by the teachers for (i) obedience, (ii) academic performance, (iii) attention, (iv) punctuality, (v) behavior with friends and (vi) behavior with teachers. The fact that the two groups were almost comparable in their results emphasized that physical exercise in some form is an important part of the curriculum for pre-adolescent children, though the findings need to be viewed with caution due to the absence of a control group.

Physical activity and yoga have different ways of influencing physical fitness, cognitive performance and self-esteem. Some of the possible mechanisms are mentioned below. Physical activity benefits physical wellbeing by changes in biological cardiovascular disease risk-factor profiles in children such as lower blood pressure, favorable lipid and lipoprotein levels and reduced adiposity [41]**.** Yoga practice favorably influences physical health in children chiefly by the effects on lung capacities, cardio-respiratory endurance [10] and the effect on muscle strength [42]. With regard to cognition, structural MRI data showed that changes in bilateral putamen volumes of the dorsal striatum and globus pallidus predicted flanker task performance at initial testing and after a year of increased physical exercise [43]. Neuroimaging studies conducted on adult yoga practitioners showed increased blood flow to the dorsolateral prefrontal cortex [44]. Hence for cognitive tasks in both physical exercise and yoga practice there seem to be specific changes in particular parts of the brain.

Physical activity and yoga also separately improved emotional wellbeing in youth [8,18]. The mechanisms underlying these benefits have not been clearly worked out and may involve complex neuro-chemical changes and modified functioning of brain areas within the limbic circuit. These are possible areas for future study.

The main limitations of the present study are given below:

(i) The study included two independent groups. The school would not permit a third group who had no intervention during school hours hence there was no control group. (ii) The yoga and physical exercise programs had to fit in with the regular school schedule. This was the main reason why the follow up period was limited to 3 months. Every 4 months during the school year children have examinations and both before and after the examinations there would have been gaps during which they would not have practiced yoga or physical exercise regularly. (iii) The Eurofit Physical Fitness Test Battery actually has ten items, but since the testing was already time-consuming, the 20 m endurance shuttle-run was omitted. The sit-and-reach test was completed but since there were errors in the way data were collected, these data were not analyzed. (iv) Since the participants in this study were geographically localized to the north of India, the generalizability of the findings needs to be further investigated in a sample drawn from diverse geographical and cultural backgrounds.

Despite these limitations the present randomized controlled trial demonstrated that measures of physical fitness, cognition and teacher rated performance and behavior improved following yoga and physical exercise in school children, when 3 month data were compared with baseline data. Social self-esteem improved after physical exercise compared to yoga. These are the possible effects of the two interventions, with a degree of uncertainty due to the absence of a control group.

## Competing interests

The authors declare that they have no competing interests.

## Authors’ contributions

ST designed the study, interpreted the results and compiled the manuscript; NS participated in designing the study, data collection and analysis and in compiling the manuscript; AKB participated in data collection and data analysis and assisted in compiling the manuscript; AK assisted in data taking and in the interventions; AB designed the intervention. All authors read and approved the final manuscript.
